# Hetero-grafting affects sugar and flavonoid accumulation in ‘Misty’ blueberry (*Vaccinium corymbosum*)

**DOI:** 10.3389/fpls.2026.1995904

**Published:** 2026-10-22

**Authors:** Yaxin Wang, Lingxiang Li, Xuzhe Xiong, Shuangwen Wang, Hang Ni, Feng He, Bo Zhu, Jiaxin Xiao

**Affiliations:** Anhui Provincial Key Laboratory of Biodiversity Conservation and Ecological Security in the Yangtze River Basin, College of Life Sciences, Anhui Normal University, Wuhu, China

**Keywords:** flavonoids, grafting, sugars, transcription factors, *Vaccinium corymbosum*

## Abstract

**Introduction:**

Grafting can be used in blueberry cultivation to improve soil adaptability and scion fruit quality. However, the underlying molecular mechanisms remain poorly understood.

**Methods:**

We investigated the effects of rabbiteye blueberry (*Vaccinium virgatum*) ‘Climax’ and ‘Brightwell’ rootstocks on fruit quality, biochemical characteristics, and related gene expression in ‘Misty’ blueberry (*Vaccinium corymbosum*).

**Results:**

The fruit quality of ‘Climax’-grafted ‘Misty’ (CM) was superior to that of ‘Brightwell’-grafted ‘Misty’ (BM). CM showed greater scion stem diameter and fruit size, as well as the net photosynthetic rate, and chlorophyll contents in leaves. Notably, LC-MS/MS analysis detected significant increases in specific sugars and flavonoids in CM fruit, with glucose and fructose contents 10.2% and 11.4% higher than BM, respectively. Twenty-four out of the 31 quantified flavonoids were more abundant in CM. In contrast, 26 of the 28 detected amino acids accumulated at higher levels in BM, as did most organic acids. A total of 1,411 differentially expressed genes, including 830 upregulated and 581 downregulated genes, were detected between CM and BM fruit, in which the genes associated with sugar (*VcSWEET12* and *VcINV1*) and flavonoids (*VcF3′H* and *VcUFGT*) were upregulated in CM fruit. An integrated analysis pinpointed nine transcription factors, including *VcWRKY75* and *VcWRKY24*, as potential modulators of sugar metabolism and flavonoid biosynthesis.

**Discussion:**

These findings indicate that different rabbiteye blueberry rootstocks are associated with distinct physiological and metabolic responses in southern highbush blueberry. Compared with ‘Brightwell’, the ‘Climax’ rootstock was more conducive to improving the comprehensive quality of ‘Misty’ blueberry fruit. The results provide candidate molecular targets for dissecting the process of fruit quality formation mediated by rootstock–scion interactions, and they form a basis for high-quality blueberry cultivation.

## 1 Introduction

Blueberry (*Vaccinium* spp.) is a shrub fruit tree in the family Ericaceae. In recent years, China’s blueberry industry has developed rapidly, forming five initial major blueberry producing areas, including the Yangtze River Basin, with 27 provinces, municipalities, and autonomous regions engaged in large-scale cultivation (Li et al., 2021). At present, China ranks first in the world in blueberry planting area and total output, and the production capacity and associated economic benefits are increasing. The blueberry industry, including purchases and sales, is experiencing a period of rapid development (Li et al., 2025). Blueberry fruit quality is closely linked to the accumulation of sugars, organic acids, amino acids, anthocyanins, and flavonoids. Sugars and organic acids determine sweetness and acidity, and their balance shapes overall flavor (Zhang et al., 2022; Sun et al., 2026). Amino acids participate in fruit metabolism and flavor formation, while anthocyanins and other flavonoids govern fruit color, antioxidant capacity, and nutritional value (Zhang et al., 2023; Xie et al., 2025). Recent transcriptomic and metabolomic work has uncovered the molecular underpinnings of blueberry fruit quality. Comparative analysis of ‘Anna’ and ‘Misty’ revealed cultivar-specific sugar accumulation linked to carbohydrate metabolism (Yang et al., 2024). Multi-omics studied on blueberry cultivars with divergent fruit colors also showed substantial variation in flavonoid and anthocyanin pathways (Zhang et al., 2024). Moreover, soluble sugars and acidity shifts during ripening coincide with widespread metabolic and transcriptional changes (Cao et al., 2026). Collectively, these reports demonstrate that blueberry fruit quality variation arises from coordinated alterations in metabolite abundance and gene expression.

The climate conditions in the Wanjiang River Basin of Anhui Province are relatively suitable for the cultivation and production of rabbiteye blueberry (*V. virgatum* Ait.) and southern highbush blueberry (SHB) (*V. corymbosum* L.). Rabbiteye blueberry exhibits vigorous growth, strong adaptability, high yield, and moderate fruit quality. However, mature harvesting occurs during the rainy season, which is inconvenient and results in time-consuming storage and transportation (Pritts et al., 1992). The SHB, including ‘Misty’ blueberry, has excellent fruit quality, with a thick epicuticular wax, and moderate fruiting. In the Wanjiang River Basin, its maturity period is approximately one month earlier than that of the rabbiteye blueberry, generally maturing in mid to late May, but the tree vigor is relatively weak (Zhu et al., 2024). These complementary traits support the practical application of vigorous rabbiteye blueberry cultivars as rootstocks for SHB. Nevertheless, the impacts of rabbiteye rootstocks on SHB, especially with respect to fruit quality, remain to be further characterized.

The vitality of a rootstock can alter the balance between nutritional and reproductive growth, thereby affecting the water-use efficiency, fruit yield, and quality of grafted trees (Tomić et al., 2022; Raghavan et al., 2025). The growth vitality of SHB ‘Sharpblue’ grafted onto *V. bracteatum* is significantly enhanced, and the early fruit yield and quality are also significantly improved (Xu et al., 2014). The rootstock genotype can also affect gene expression in scions, thereby promoting anthocyanin synthesis and vitamin C (Vc) accumulation in fruit (Shahkoomahally et al., 2020; Lana et al., 2021). The rootstock in hetero-grafting promotes the synthesis and accumulation of anthocyanins in grape berries (Zhong et al., 2022). There have also been different reports that, although five types of multi-embryo rootstocks affect the tree vigor and fruit yield of mango varieties, promoting nitrogen, phosphorus, and potassium absorption, they had little effect on fruit quality (Dubey et al., 2021). Thus, rootstocks may modify fruit quality via translocation of water, minerals and other substances to the scion, yet the underlying mechanisms remain complex (Kaleem et al., 2026b). Recent metabolomic and transcriptomic research has advanced our knowledge of rootstock effects beyond fruit phenotypic traits to metabolic and transcriptional regulation. In grafted crops including tomato, cucumber, melon, and watermelon, rootstock types alter the accumulation of sugars, organic acids, amino acids, volatile and other quality-related metabolites, alongside rewired metabolic pathways and gene expression (Miao et al., 2019; Zhou et al., 2022; Kaleem et al., 2022, 2024; Zhang et al., 2026; Yang et al., 2026). Together, these findings demonstrate that rootstock-dependent variation in fruit quality originate from coordinated scion metabolic and transcriptional responses. However, omics research on blueberry fruit quality mainly focuses on cultivar variation, fruit development and ripening, or postharvest changes. While multi-omics studies of rootstock-regulated fruit quality are limited to other horticultural crops. How rabbiteye rootstocks modulate physiology and metabolism in the same SHB scion, and the associated transcriptional changes, is still unclear. In this study, ‘Misty’ grafted onto two rabbiteye blueberry rootstocks was grown under uniform field conditions. We combined physiological and fruit-quality measurements with metabolomic and transcriptomic profiling to uncover rootstock impacts on ‘Misty’, and screen candidate genes and transcription factors regulating sugar and flavonoid accumulation. This study provides physiological and molecular evidence for evaluating rabbiteye blueberry rootstocks, and offers practical guidance for rootstock selection and high-quality SHB cultivation in the Wanjiang River Basin.

## 2 Materials and methods

### 2.1 Plant materials

Fruit and leaves were collected in May of 2024 and 2025 at the blueberry orchard of Anhui Huiking Agricultural Co., Ltd., Bailin Village, Nanling County, Anhui Province (30°51’45.71’’N, 118°23’38.13’’E), and the permission was obtained from the company. The experimental materials were SHB ‘Misty’ grafted onto rabbiteye blueberry ‘Climax’ (CM) and ‘Brightwell’ (BM). ‘Climax’ and ‘Brightwell’ bushes were planted in the autumn of 2011, with a plant spacing of 1 m × 2 m on a red-yellow loam soil having the following characteristics: pH 4.8, organic matter 24.8 g kg^-1^, total nitrogen 1.2 g kg^-1^, total phosphorus 0.3 g kg^-1^, total potassium 6.6 g kg^-1^, hydrolyzable nitrogen 156.5 mg kg^-1^, available phosphorus 80 mg kg^-1^, and available potassium 103.2 mg kg^-1^. Each plant was selected with 3–4 main stems for grafting in March 2023, with the grafting interface at approximately 20 cm above the ground.

The experimental plot was arranged as a randomized complete block design with CM and BM, three blocks and three plants within each block, resulting in nine plants per rootstock-scion combination and 18 plants in total. Mature fruit were collected on May 26, 2024 and May 27, 2025 based on the appearance of similar blue-black colors on the fruit surface. Approximately 270 mature fruits were harvested from each block. Approximately 30% of the fruits were frozen in liquid nitrogen and brought to the laboratory for storage at -80 °C for later use, others were brought to the laboratory in an ice box and stored in a refrigerator at 4 °C for physiological index determination.

### 2.2 Fruit quality traits

The fresh fruit were weighed, the longitudinal and transverse diameters were measured using a Vernier caliper, the hardness levels were measured using a handheld hardness tester, the total soluble solids contents were measured using a WZB35 handheld sugar meter, titratable acidity levels were determined by the potentiometric titration (Li et al., 2023a), the ratios of total soluble solids to titratable acidity (solid:acid ratio) were calculated, the Vc contents were determined using UV spectrophotometer, and the anthocyanin contents were determined using the pH differential method (Song et al., 2013; Feng, 2016).

The heights from the exposed soil surfaces to the grafting unions were measured, and the diameters of the main trunks at 5 cm above and 5 cm below the grafting unions, which represent the thicknesses of the scion and rootstock, respectively, were measured using a Vernier caliper. The fruit yields (g plant-1) were obtained by weighing the harvested fruit. Fuzzy comprehensive evaluations of fruit quality were conducted in accordance with the methods reported by Feng (2016) and Zhu et al. (2024). Trait weights are listed in **Supplementary Table S3**, and the comprehensive evaluation value was calculated as the weighted sum of normalized trait values. Principal component analysis (PCA) used Z-score standardization and extracted components with eigenvalues > 1.

### 2.3 Photosynthetic parameters and mineral elements

On 26 April 2025, from 8:30 a.m. to 11:30 a.m., the Li-6400XT portable photosynthesis measurement system (LI-COR, USA) was used to measure photosynthetic parameters, such as net photosynthetic rate (Pn), transpiration rate (Tr), stomatal conductance (Gs), and intercellular CO2 concentration (Ci) of the SHB blueberry grafted onto the two rootstocks, in accordance with the method reported by Zhu et al. (2024). On 26 April 2025, approximately 30 leaves were collected from each rootstock–scion combination and brought to the laboratory in an ice box. Approximately 30% of the leaves were extracted with 95% ethanol to determine the contents of chlorophyll and carotenoids, and others were digested to determine the contents of mineral elements using an inductively coupled plasma mass spectrometer (PerkinElmer NexION® 1000G) in accordance with Zhu et al. (2024).

### 2.4 Sugars and organic acids

To determine the contents of sugars and organic acids in fruit, an extraction solution was prepared in accordance with the method reported by Gao et al. (2025). Sugars were analyzed using an Agilent 1260 Infinity III high-performance liquid chromatograph (Agilent, USA) and an evaporative light scattering detector equipped with a Waters XBridge Amide column (4.6 × 150 mm, 5 μm) at a column temperature of 35 °C. The mobile phases were Phase A (30% acetonitrile aqueous solution) and Phase B (80% acetonitrile aqueous solution). The gradient elution procedure was as follows: 0–10 min: 0% A, 100% B; 10–23 min: 0%–50% A, 100%–50% B; 23–23.1 min: 50%–0% A, 50%–100% B; 23.1–26 min: 0% A, 100% B. The flowrate was 1 mL min-1, and the injection volume was 10 μL. The contents of mixed standard solutions were 12.5, 25, 50, 100, 200, 400, and 800 μg mL-1.

Organic acids were analyzed using an ultra-high performance liquid chromatography tandem mass spectrometer (LC-MS/MS) (Qsight LX 50, Qsight 420, PerkinElmer, USA) and separated using Waters ACQUITY UPLC HSS T3 column (2.1 × 100 mm, 1.8 μm) at a column temperature of 30 °C. The mobile phases were Phase A (water–0.1% formic acid) and Phase B (100% acetonitrile), with the following gradient elution procedure: 0–2 min: 99% A, 1% B; 2–6 min: 99%–0% A, 1%–100% B; 6–8 min: 0% A, 100% B; 8–8.1 min: 0%–99% A, 100%–1% B; 8.1–10 min: 99% A, 1% B. The flowrate was 0.3 mL/min, and the injection volume was 1 μL. The contents of oxalic acid standard solutions were 50, 100, 500, 1,000, 5,000, 10,000, and 50,000 ng/mL, and contents of mixed standard solutions of other organic acids were 5, 10, 50, 100, 500, 1,000, and 5,000 ng mL-1.

### 2.5 Amino acids and flavonoids

The extraction solutions for determining contents of amino acids and flavonoids in fruit were prepared in accordance with Gao et al. (2025). Amino acids were analyzed using an ultra-high performance liquid chromatography tandem mass spectrometer (Xevo™ TQ-S, Waters Corporation, USA) equipped with Agilent InfinityLab Poroshell 120 HILIC-Z chromatographic column (2.1 × 100 mm, 2.7 μm). The column temperature was 35 °C. The mobile phases were Phase A (10 mM ammonium formate, adjusted to pH = 3 with formic acid) and Phase B (10 mM ammonium formate -90% acetonitrile solution, adjusted to pH = 3 with formic acid). The gradient program was as follows: 0–2 min: 5% A, 95% B; 2–4 min: 5%–20% A, 95%–80% B; 4–6 min: 20%–45% A, 80%–55% B; 6–12 min: 45% A, 55% B; 12–12.1 min: 45%–5% A, 55%–95% B; 12.1–15 min: 5% A, 95% B. The flowrate was 0.3 mL min ^-1^, and the injection volume was 1 μL. The individual standard stock solutions of 16 amino acids (cysteine, tryptophan, asparagine, glycine, glutamine, gamma aminobutyric acid, S-adenosylmethionine, S-adenosylhomocysteine, homocysteine, theanine, hydroxyproline, citrulline, hydroxylysine, kynurenine, taurine, and β-alanine) were each diluted with a dilution of a 1 mM HCl-20% ethanol solution to final concentrations of 10, 25, 50, 250, 500, 2,500, and 5,000 ng/mL. The mixed standard stock solutions of 15 other amino acids were each diluted to final concentrations of 0.1, 0.25, 0.5, 2.5, 5, 25, and 50 nmol mL^-1^, which were converted from mass-based concentrations according to the respective molecular weight of each amino acid.

Flavonoid contents were determined using an ultra-high performance liquid chromatography tandem triple quadrupole mass spectrometer (LC-MS/MS, Xevo™ TQ-S, Waters Corporation, USA) equipped with an Agilent poroshell 120 EC-C 18 chromatographic column (2.1 × 150 mm, 2.7 μm). The column temperature was 40 °C. The mobile phase consisted of two solvents: Solvent A, 0.1% formic acid aqueous solution and Solvent B, acetonitrile. The gradient program was as follows: 0–1 min: 90% A, 10% B; 1–2 min: 90%–80% A, 10%–20% B; 2–5 min: 80%–20% A, 20%–80% B; 5–6 min: 20%–5% A, 80%–95% B; 6–9 min: 5% A, 95% B; 9–9.1 min: 5%–90% A, 95%–10% B; 9.1–12 min: 90% A, 10% B. The flowrate was 0.3 mL/min, and the injection volume was 2 µL. The standard flavonoid solution concentrations were 2, 5, 20, 50, 200, 500, and 2,000 ng mL ^-1^.

### 2.6 Transcriptome sequencing and data analysis

RNA from mature fruit was extracted using a modified combined CTAB-pBIOZOL extraction protocol. The obtained RNA was dissolved in diethyl pyrocarbonate (DEPC) -treated water, and samples were quantified and their quality assessed using a Bioanalyzer. mRNA library construction was performed and reads were sequenced on an MGI high-throughput sequencing platform by Metware Biotechnology Co., Ltd. (Wuhan, China). Raw reads were preprocessed with fastp v0.23.2 to remove adapter sequences and low-quality reads. Read pairs were filtered out if either read contained > 10% ambiguous or over 50% of bases had a Phred quality scores ≤ 20. Clean reads were aligned to the *Vaccinium_corymbosum*_v1.0 reference genome using HISAT2 v2.2.1. Gene-level raw read counts were obtained via featureCounts v2.0.3, and gene expression was quantified as fragments per kilobase of transcript per million mapped fragments (FPKM) with StringTie v2.1.6. PCA was performed to assess sample relationships and the reproducibility of biological replicates. Differential expression analysis between CM and BM groups was analyzed using DESeq2 v1.22.1 based on raw gene read counts. Genes with |log_2_FC| > 0.585 (FC > 1.5 or FC < 0.667) and *p* < 0.05 were defined as differentially expressed genes (DEGs) (Olmedo Cuaspud et al., 2025). Given that the comparison was performed on the same scion genotype grafted onto two different rootstocks under field conditions, these thresholds were adopted to retain moderate yet biologically relevant transcriptional alterations. Plant transcription factors (TFs) were identified and categorized using iTAK. Gene Ontology (GO) annotation and Kyoto Encyclopedia of Genes and Genomes (KEGG) pathway enrichment analyses for DEGs were performed using clusterProfiler software.

### 2.7 Weighted gene co-expression network analysis

To screen for key genes, we used a weighted gene co-expression network analysis (WGCNA) to divide DEGs into modules. First, low-expression genes were filtered out to ensure the reliability of network construction. Then, a soft thresholding power of 18 was selected, the minimum number of genes per module was set to 50, and a module eigengene distance threshold of 0.25 was used to merge highly similar modules.

### 2.8 Analysis of promoter cis-acting elements

The key genes identified in this study were selected. The 2,000-bp genomic sequences upstream of the transcription start sites from the reference genome database were extracted as the promoter regions and submitted to the PlantCARE online database for a cis-acting element analysis. Finally, TBtools software was used for visualization.

### 2.9 Validation of gene expression by qRT-PCR

In total, 20 genes related to sugar, organic acid, and flavonoid metabolisms were selected and analyzed using quantitative real-time reverse-transcription PCR (qRT-PCR). Three biological replicates were included for each rootstock-scion combination with three technical replicates per biological sample. All the primers are listed in **Supplementary Table S1**. *GAPDH* was selected as the reference gene owing to its stable expression across all samples, validated by consistent cycle threshold (Ct) values (standard deviation < 0.5) and geNorm/NormFinder analyses. The amplification efficiency of each primer pair was determined using a standard curve generated from 10-fold serial cDNA dilutions; efficiencies ranged from 95% to 105% with R² > 0.99. Relative gene expression levels were calculated using the 2^-ΔΔCT^ method (LLivak and Schmittgen, 2001). The qRT-PCR thermal profile was: initial denaturation at 95 °C for 30 s, followed by 40 cycles of 95 °C for 5 s and 60 °C for 30 s. Each sample’s qRT-PCR analysis was performed in triplicate.

### 2.10 Statistical analyses

The data obtained from the measurement were analyzed using Microsoft EXCEL software, and both the PCA and correlation analysis were performed using SPSS Statistics 27.0. Two-way analysis of variance (ANOVA) was used to evaluate the effects of rootstock–scion combination, year, and their interaction, followed by Tukey’s test for multiple comparisons. Independent-samples t-tests were used for comparisons between two groups. Significant differences were determined at p < 0.05.

## 3 Results

### 3.1 Growth and fruit quality

There was no significant difference in tree height, canopy width, rootstock height, rootstock thickness, or yield between CM and BM, but the scion diameter of CM was significantly greater than that of BM (**Supplementary Table S2**). As shown in **Table 1**, fresh weight and both longitudinal and transverse diameters were significantly greater in CM than in BM, but there was no significant difference in fruit hardness or in fruit shape index between CM and BM. Additionally, fresh weight and both longitudinal and transverse diameters of fruit in 2025 were significantly less than in 2024, and the fruit shape had changed from flat spherical to spherical.

**Table 1:** Fresh weight, hardness, and fruit size in mature fruit of ‘Misty’ blueberry grafted onto ‘Climax’ (CM) and ‘Brightwell’ (BM).

Rootstock-scion combination	Fresh weight (g/fruit)	Hardness (kg·cm^-2^)	Longitudinal diameter (mm)	Transverse diameter (mm)	Fruit shape index
CM	2.23 ± 0.05*	0.85 ± 0.13	12.76 ± 0.01*	16.07 ± 0.2*	0.80 ± 0.01
BM	1.73 ± 0.03	0.74 ± 0.01	11.99 ± 0.28	15.16 ± 0.06	0.79 ± 0.02
			Year		
2024	2.26 ± 0.03*	0.76 ± 0.03	13.22 ± 0.18*	17.22 ± 0.12*	0.77 ± 0.01
2025	1.75 ± 0.02	0.9 ± 0.02*	11.58 ± 0.04	14.01 ± 0.03	0.83 ± 0.00*
			Interaction		
CM×2024	2.6 ± 0.07 a	0.83 ± 0.05 a	13.82 ± 0.14 a	18.00 ± 0.44 a	0.77 ± 0.01 b
CM×2025	1.87 ± 0.16 b	0.87 ± 0.23 a	11.7 ± 0.14 c	14.15 ± 0.06 c	0.83 ± 0.01 a
BM×2024	1.92 ± 0.01 b	0.69 ± 0.02 a	12.62 ± 0.49 b	16.45 ± 0.23 b	0.77 ± 0.02 b
BM×2025	1.63 ± 0.13 c	0.93 ± 0.27 a	11.46 ± 0.14 c	13.87 ± 0.12 c	0.83 ± 0.01 a

Data are mean ± standard deviation(n=3). Different lowercase letters in the same column indicate significant difference among rootstock × year interactions according to two-way ANOVA followed by Tukey’s test (*p* < 0.05). *indicates significant difference between CM and BM within the same year according to independent-samples t-test (p < 0.05).

Total soluble solids and Vc contents, as well as the solid:acid ratio, of CM were significantly greater than those of BM, but the CM titratable acidity was significantly less than that of BM. Vc and anthocyanin contents in 2025 were significantly greater than in 2024; however, titratable acidity, total soluble solids content, and the solid:acid ratio in 2024 were significantly greater than in 2025 (**Table 2**).

**Table 2:** Titratable acidity, total soluble solids, solid: acid ratio, vitamin C, and anthocyanin in mature fruit of ‘Misty’ blueberry grafted onto ‘Climax’ (CM) and ‘Brightwell’ (BM).

Rootstock-scion combination	Titratable acidity (%)	Total soluble solids (%)	Solid:acid ratio	Vitamin C (mg/100g)	Anthocyanin (mg/100g)
CM	0.70 ± 0.01	10.98 ± 0.23*	15.65 ± 0.26*	231.14 ± 18.64*	75.89 ± 3.89
BM	0.83 ± 0.01*	9.95 ± 0.18	12.06 ± 0.21	144.65 ± 2.64	81.16 ± 6.62
Year					
2024	0.81 ± 0.00*	11.22 ± 0.06*	14.05 ± 0.05*	108.11 ± 6.71	55.43 ± 3.61
2025	0.72 ± 0.00	9.72 ± 0.20	13.66 ± 0.40	267.67 ± 22.77*	104.05 ± 4.25*
Interaction					
CM×2024	0.73 ± 0.03 b	11.87 ± 0.38 a	16.19 ± 0.21 a	100.98 ± 17.36 c	61.26 ± 15.87 c
CM×2025	0.67 ± 0.02 c	10.10 ± 0.20 b	15.11 ± 0.71 b	361.29 ± 51.83 a	90.51 ± 15.89 b
BM×2024	0.89 ± 0.03 a	10.57 ± 0.32 b	11.92 ± 0.28 c	115.23 ± 4.03 c	49.61 ± 14.49 c
BM×2025	0.77 ± 0.02 b	9.33 ± 0.21 c	12.21 ± 0.16 c	174.06 ± 8.72 b	117.6 ± 9.26 a

Data are mean ± standard deviation (n = 3). Different lowercase letters in the same column indicate significant difference among rootstock × year interactions according to two-way ANOVA followed by Tukey’s test (*p* < 0.05). *indicates significant difference between CM and BM within the same year according to independent-samples t-test (p < 0.05).

The weight values of quality traits provided by Zhu et al. (2024), with slight modifications, are shown in **Supplementary Table S3**. The comprehensive evaluation values are shown in **Supplementary Table S4**. The fruit quality of CM was good (with a comprehensive evaluation value of 0.45–0.65), whereas the fruit quality of BM was moderate (with a comprehensive evaluation value of 0.25–0.45). A PCA was conducted on nine fruit quality traits. The results are shown in **Supplementary Table S5**. The cumulative variance contribution rate of the first two principal components reached 90.007%. The principal component scores were ordered sequentially as CM and BM (**Supplementary Table S6**).

### 3.2 Photosynthetic parameters and mineral elements

The *P_n_*, *G_s_*, *C_i_*, and *T_r_* of CM were significantly greater than those of BM (**Table 3**). *P_n_* showed a significant positive correlation with *G_s_*, *C_i_*, and *T_r_*; *G_s_* showed a significant positive correlation with *C_i_* and *T_r_*; and *C_i_* was significantly positively correlated with *T_r_* (**Supplementary Table S7**). The contents of chlorophyll a, chlorophyll b, and chlorophyll a+b in CM were significantly higher than those in BM, but no significant differences were observed in carotenoid content or chlorophyll a/b ratio between CM and BM (**Table 3**). The potassium (K), calcium (Ca), iron (Fe), manganese (Mn), copper (Cu), and zinc (Zn) contents in CM leaves were significantly greater than in BM leaves. There was no significant difference in magnesium (Mg) or phosphorus (P) contents between CM and BM, but the boron(B) content of BM was significantly greater than that of CM (**Supplementary Table S8**).

**Table 3:** Photosynthetic parameters and pigments in leaves of ‘Misty’ blueberry grafted onto ‘Climax’ (CM) and ‘Brightwell’ (BM).

Photosynthetic parameters	CM	BM	Photosynthetic pigments	CM	BM
*P_n_*(μmol·m^-2^·s^-1^)	15.54 ± 0.86*	13.82 ± 0.63	Chlorophyll a (mg/g)	1.51 ± 0.03*	1.34 ± 0.03
*G_s_*(mol·m^-2^·s^-1^)	0.16 ± 0.02*	0.10 ± 0.01	Chlorophyll b (mg/g)	1.01 ± 0.02*	0.87 ± 0.03
*C_i_*(μmol·mol^-1^)	240.00 ± 14.88*	157.80 ± 22.8	Chlorophyll a+b (mg/g)	2.52 ± 0.02*	2.21 ± 0.02
*T_r_*(mmol·m^-2^·s^-1^)	2.73 ± 0.21*	1.51 ± 0.10	Carotenoid (mg/g)	0.23 ± 0.00	0.22 ± 0.00
			Chlorophyll a/b	1.50 ± 0.05	1.54 ± 0.07

Data are mean ± standard deviation (n=3). * indicate significant difference between CM and BM according to independent-samples t-test (*p* < 0.05).

### 3.3 Sugars, organic acids, amino acids, and flavonoids

In total, 4 sugars, fructose, glucose, sucrose, and maltose, and 12 organic acids, were detected in mature fruit of CM and BM. The levels of the four detected sugars were significantly higher in CM fruit than in BM fruit (**Table 4**).

**Table 4:** Contents of sugars and organic acids in mature fruit of ‘Misty’ blueberry grafted onto ‘Climax’ (CM) and ‘Brightwell’ (BM).

Sugars	CM	BM	Organic acids	CM	BM
glucose(mg/g)	61.29 ± 0.58*	55.61 ± 1.37	citric acid(μg/g)	9368.25 ± 346.45	11152.81 ± 253.78*
fructose(mg/g)	53.21 ± 1.04*	47.76 ± 0.70	quinic acid(μg/g)	812.16 ± 31.45	953.92 ± 17.73*
sucrose(mg/g)	6.57 ± 0.16*	5.35 ± 0.07	maleic acid(μg/g)	175.42 ± 9.55	205.62 ± 3.47*
maltose(mg/g)	0.09 ± 0.00*	0.06 ± 0.00	malic acid(μg/g)	170.29 ± 4.63	199.99 ± 3.89*
			malonic acid(μg/g)	1.18 ± 0.04	1.45 ± 0.02*
			lactic acid(μg/g)	0.47 ± 0.02	0.63 ± 0.01*
			tartaric acid(μg/g)	1.90 ± 0.06*	1.59 ± 0.02
			oxalic acid(μg/g)	61.37 ± 2.71	59.63 ± 1.45
			shikimic acid(μg/g)	48.18 ± 1.79	47.92 ± 1.68
			acetic acid(μg/g)	9.54 ± 0.42	9.04 ± 0.14
			succinic acid(μg/g)	4.71 ± 0.39	4.30 ± 0.23
			fumaric acid(10^-3^μg/g)	95.38 ± 3.40	102.62 ± 1.38*

Data are mean ± standard deviation (n=3). *indicate significant difference between CM and BM according to independent-samples t-test (*p* < 0.05).

Among the detected organic acids, the oxalic acid, shikimic acid, acetic acid, and succinic acid contents showed no significant differences between CM and BM. The tartaric acid content in CM fruit was significantly greater than in BM fruit, whereas the contents of seven other organic acids were significantly greater in BM fruit than in CM fruit (**Table 4**).

In total, 28 amino acids were detected in mature fruit. Among them, the taurine content in CM fruit was significantly greater than in BM fruit. No significant difference was found in kynurenine between CM and BM, and the other 26 amino acids were significantly higher in BM than in CM (**Table 5**). A total of 31 flavonoids were detected. No significant difference was detected in the content of bergapten, psoralen dihydroflavonoid, or apigenin between CM and BM. The contents of nobiletin, protocatechuic acid, umbelliferone, and neohesperidin dihydrochalcone in BM were significantly greater than in CM, while the levels of the other 24 flavonoids were significantly higher in CM than in BM (**Table 5**).

**Table 5:** Contents of amino acids (μg/g) and flavonoids (ng/g) in mature fruit of ‘Misty’ blueberry grafted onto ‘Climax’ (CM) and ‘Brightwell’ (BM).

Amino acids	CM	BM	Flavonoids	CM	BM
threonine	18.74 ± 0.24	37.45 ± 0.31*	hyperoside	14163.61 ± 179.42*	10041 ± 130.25
valine	16.75 ± 0.19	24.16 ± 0.26*	catechin	14027.03 ± 55.99*	11437.00 ± 203.82
histidine	11.01 ± 0.05	24.56 ± 0.23*	quercetin	3982.92 ± 40.51*	3346.92 ± 40.42
tryptophan	9.02 ± 0.02	33.55 ± 0.25*	epicatechin	3970.52 ± 52.58*	2558.84 ± 36.48
lsoleucine	7.21 ± 0.04	8.74 ± 0.01*	chlorogenic acid	1988.12 ± 17.52*	987.96 ± 11.87
lysine	3.03 ± 0.04	7.31 ± 0.04*	rutin	1385.72 ± 16.68*	984.28 ± 18.83
leucine	2.64 ± 0.10	4.17 ± 0.01*	protocatechualdehyde	86.61 ± 0.22*	73.07 ± 0.44
phenylalanine	2.29 ± 0.02	2.96 ± 0.07*	gallic acid	51.87 ± 0.55*	39.18 ± 0.38
glutamic acid	182.45 ± 1.22	285.08 ± 1.82*	hispidulin	41.32 ± 0.47*	37.16 ± 0.23
arginine	130.75 ± 1.00	198.87 ± 3.09*	aurantio-obtusin	31.20 ± 0.96*	15.60 ± 0.13
aspartic acid	81.91 ± 0.55	129.14 ± 2.07*	ginkgetin	29.48 ± 0.22*	14.88 ± 0.49
glutamine	56.04 ± 0.31	106.04 ± 1.22*	rhein	16.02 ± 0.41*	9.20 ± 0.24
alanine	52.47 ± 0.44	88.34 ± 1.36*	tiliroside	23.13 ± 0.84*	14.47 ± 0.61
serine	46.43 ± 0.27	68.81 ± 0.31*	tricin	12.03 ± 0.94*	3.95 ± 0.14
gamma-aminobutyric acid	45.56 ± 0.28	99.69 ± 1.20*	naringenin	11.99 ± 0.19*	11.39 ± 0.07
asparagine	23.51 ± 0.29	48.65 ± 0.50*	2’-Hydroxychalcone	11.08 ± 0.65*	7.21 ± 0.77
tyrosine	3.18 ± 0.03	5.38 ± 0.07*	mangiferin	7.91 ± 0.06*	6.15 ± 0.23
proline	4.67 ± 0.03	21.44 ± 0.24*	kaempferol-3-O-rutinoside	7.86 ± 0.17*	3.77 ± 0.19
glycine	2.37 ± 0.02	3.91 ± 0.04*	genistin	5.97 ± 0.22*	5.14 ± 0.05
methionine	2.95 ± 0.07	4.68 ± 0.03*	baicalin	5.85 ± 0.16*	3.18 ± 0.03
S-adenosylmethionine	1.86 ± 0.02	3.16 ± 0.07*	corylifolinin	3.94 ± 0.53*	1.32 ± 0.29
citrulline	0.83 ± 0.00	6.52 ± 0.10*	psoralen	3.81 ± 0.21*	3.34 ± 0.17
cysteine	0.49 ± 0.01	0.51 ± 0.01*	loureirin B	3.49 ± 0.45*	2.37 ± 0.19
β-Alanine	0.27 ± 0.00	0.49 ± 0.01*	Vitexin-2’’-O-rhamnoside	2.33 ± 0.04*	1.67 ± 0.07
S-adenosylhomocysteine	0.15 ± 0.00	0.18 ± 0.00*	protocatechuic acid	60.53 ± 1.97	78.41 ± 0.90*
hydroxyproline	0.01 ± 0.00	0.05 ± 0.00*	neohesperidin dihydrochalcone	11.72 ± 0.19	16.84 ± 1.03*
taurine	0.14 ± 0.00*	0.13 ± 0.00	nobiletin	2.25 ± 0.09	20.70 ± 1.12*
kynurenine	0.22 ± 0.00	0.23 ± 0.00	umbelliferone	9.41 ± 0.48	15.64 ± 0.33*
			apigenin	1.53 ± 0.14	1.38 ± 0.20
			bergapten	1.31 ± 0.42	1.01 ± 0.16
			psoralen dihydroflavonoid	0.52 ± 0.11	0.64 ± 0.04

Data are mean ± standard deviation (n=3). *indicate significant difference between CM and BM according to independent-samples t-test (*p* < 0.05).

### 3.4 Transcriptome analysis

A total of 50.27 Gb of clean data were obtained. The valid data for each sample ranged from 7.88 to 9.24 Gb, the Q30 base percentage was between 97.27% and 97.62%, and the average GC content was 46.38% (**Supplementary Table S9**). The PCA plot showed that the contribution rates of the first and second principal components were 21.13% and 19.98%, respectively. Significant differences were observed between CM and BM mature fruit, and the three biological replicates within each group clustered tightly (**Figure 1A**). A total of 1,411 DEGs were identified between CM and BM, with 830 being upregulated and 581 being downregulated (**Figure 1B**). The transcription factor (TF) analysis identified 70 TF genes distributed across 29 families. The most abundant families were AP2/ERF-ERF, MYB and MYB-related, and WRKY, with 38 being upregulated and 32 being downregulated (**Figure 1C**).

**Figure 1:**
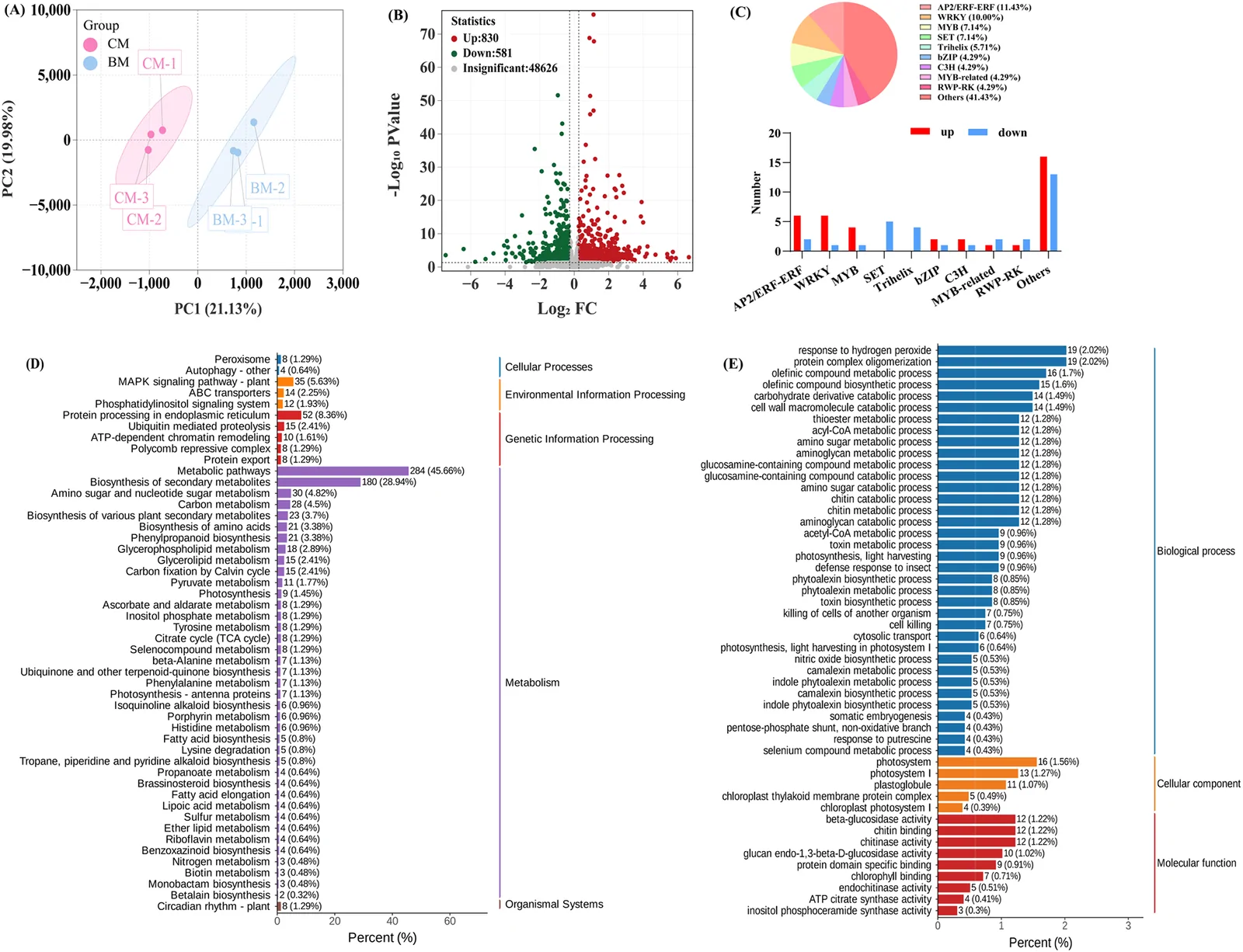
Differentially expressed genes (DEGs) in mature fruit of ‘Misty’ blueberry grafted onto ‘Climax’ (CM) and ‘Brightwell’ (BM). **(A)** PCA analysis; **(B)** Volcano plot comparison between CM and BM; **(C)** Overview of transcription factors; **(D)** KEGG pathway analysis; **(E)** GO enrichment analysis.

To investigate the biological functions of DEGs between CM and BM mature fruit, GO and KEGG pathway enrichment analyses were conducted (**Figure 1D, E**). The results revealed the significant enrichment of phenylpropanoid biosynthesis (21 genes, 3.38%), an upstream core pathway for flavonoids and anthocyanins. Additionally, significant enrichments of core pathways, including carbon metabolism (28 genes, 4.50%) and pyruvate metabolism (11 genes, 1.77%), together with the differential expression of genes involved in glycolysis and the tricarboxylic acid (TCA) cycle, were closely linked to the accumulation of sugars and organic acids.

A combined transcriptome and metabolome analysis of CM and BM revealed that differences in the flavonoid metabolic pathway were mainly concentrated in downstream modification and branch selection (**Figure 2**). For clarity, the heatmap values were horizontally standardized using the z-score method. In total, 10 differences involving DEGs, belonging to 7 gene families, were identified. No significant differences were found in the expression of structural genes, including *PAL*, *C4H*, *F3H*, and *FLS*, between CM and BM, but *CHI* was significantly upregulated, promoting the conversion of naringenin chalcone to naringenin, and the naringenin content was significantly increased, in CM. The *F3’H* gene was also significantly upregulated, promoting the conversion of kaempferol to quercetin, which led to a remarkable accumulation of quercetin. Additionally, glycosyltransferase genes, such as *UFGT* and *RhaT*, were upregulated, catalyzing the glycosylation modification of flavonols, including quercetin and kaempferol, resulting in abundant accumulations of flavonol glycosides, such as kaempferol 3-O-rutinoside, hyperoside, and rutin. In the flavonoid biosynthetic pathway, the expression levels of *LAR* and *ANR* were significantly upregulated, which may promote the biosynthesis of proanthocyanidin monomers such as catechin and epicatechin.

**Figure 2:**
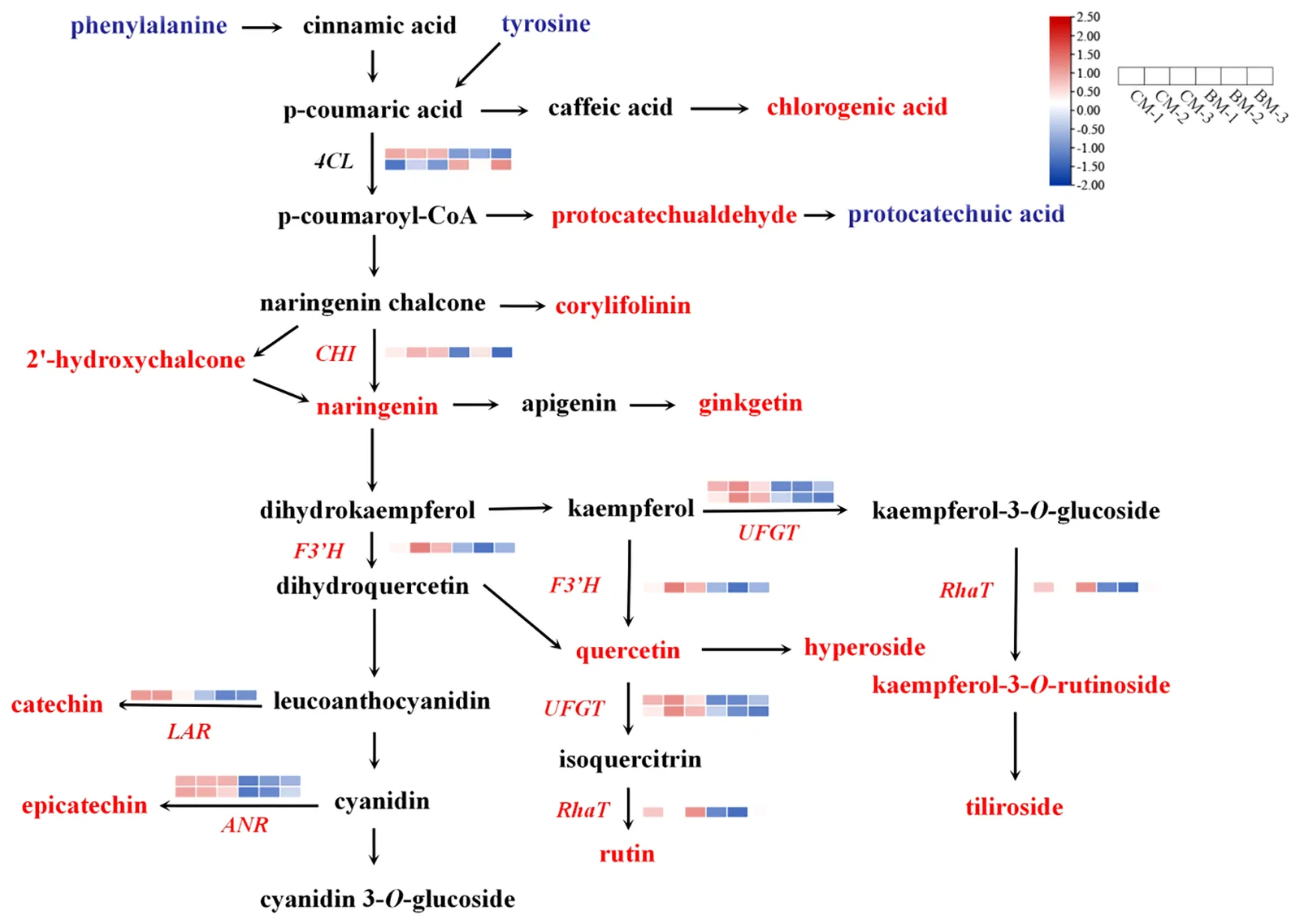
Flavonoid biosynthetic pathway in mature fruit of ‘Misty’ blueberry grafted onto ‘Climax’ (CM) and ‘Brightwell’ (BM). 4CL, 4-coumarate CoA ligase; CHI, chalcone isomerase; F3’H, flavonoid 3’-hydroxylase; LAR, leucoanthocyanidin reductase; ANR, anthocyanidin reductase; UFGT, flavonoid 3-O-glucosyltransferase; RhaT, rhamnosyltransferase. Red fonts indicate upregulated gene expression or increased metabolite accumulation in CM compared to BM, and blue fonts denote downregulation or reduced metabolite levels. The heatmap colors indicate the degree of regulation, with red representing upregulation, blue representing downregulation.

In the carbohydrate metabolism pathway, the *SWEET* and *INV* genes were significantly upregulated. This accelerated sugar transport and sucrose decomposition, leading to significant accumulations of sucrose, glucose, and fructose in CM fruit. In the TCA cycle, the significant downregulation of *PDH* and upregulation of *ACL* were found, as well as the decreased contents of citric acid, in CM. In addition, the downregulated expression of *ACC* limited malonic acid biosynthesis (**Supplementary Figure S1**). These results indicated that, compared with BM, CM fruit exhibited elevated carbohydrate levels and reduced organic acid contents by promoting sucrose decomposition and hexose accumulation while inhibiting key steps in the TCA cycle.

### 3.5 WGCNA and association analysis

To explore the relationship between gene co-expression modules and metabolite accumulation, a WGCNA was performed on DEGs and quality traits, including contents of flavonoids, sugars, and organic acids, and a module–trait correlation network was constructed, yielding five co-expression modules (**Figures 3A, B**). The turquoise module was strongly positively correlated with various flavonoids, such as epicatechin, catechin, quercetin, and rutin, as well as sugars, including glucose and fructose, and it was strongly negatively correlated with malic acid and citric acid. The blue module was significantly positively correlated with phenylalanine, malic acid, and citric acid. Additionally, DEGs involved in flavonoid, sugar, and organic acid metabolism pathways identified in previous analyses were compared with the genes enriched in each module. Most DEGs related to flavonoid, sugar, and organic acid metabolisms were enriched in the turquoise module (17 genes), with small numbers in the blue (5 genes), yellow (2 genes), and green (2 genes) modules. Thus, the turquoise module was selected for further investigation.

**Figure 3:**
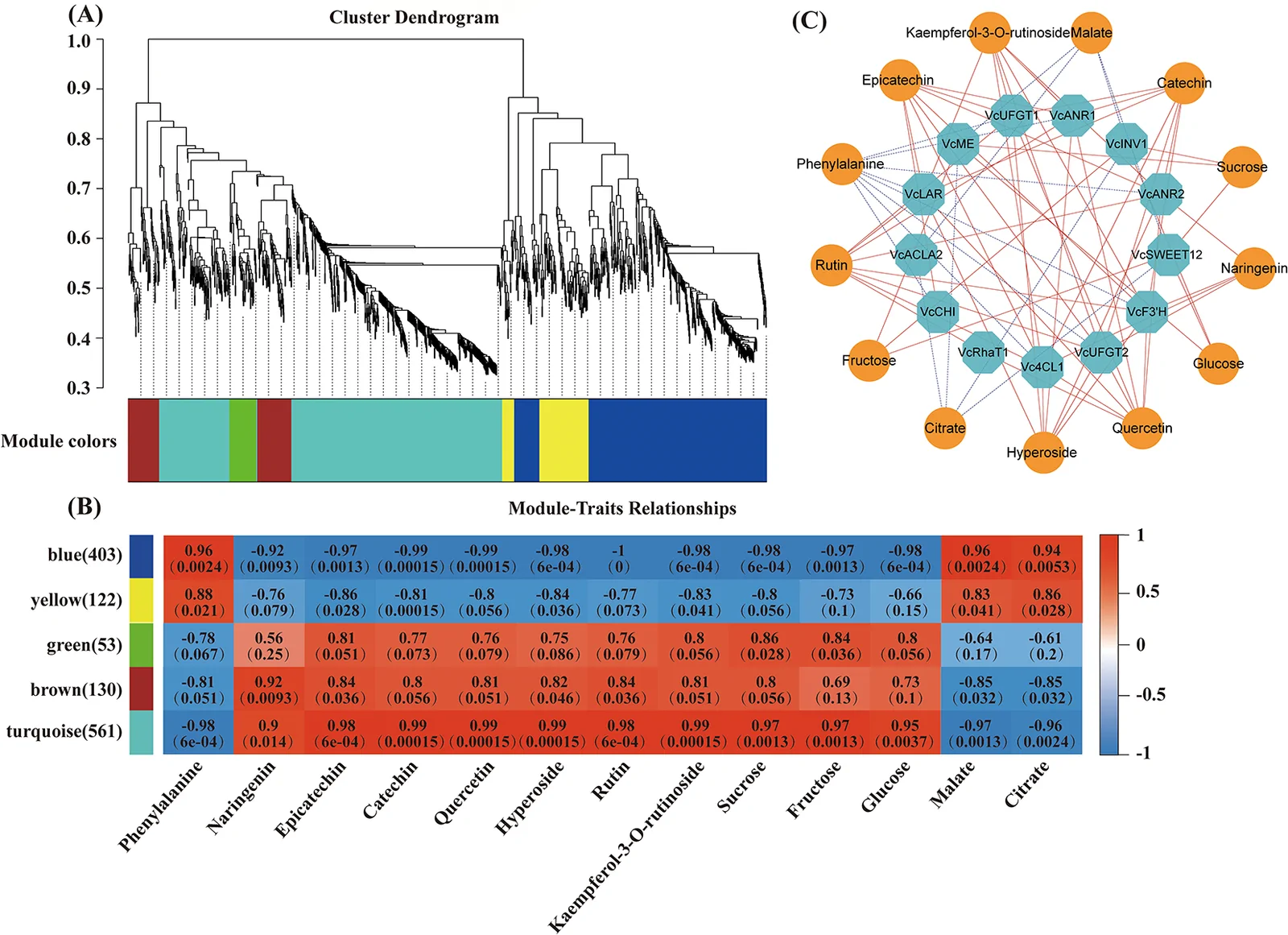
WGCNA and correlation analysis of transcriptomic and metabolomic data. **(A)** Gene dendrogram and module colors. The colored bars indicate module assignment, with each color representing a distinct coexpression module. **(B)** Module-metabolite correlation heatmap. Each row corresponds to a module, and each column represents a trait. The numbers in each cell indicate the Pearson correlation coefficient between the module eigengene and the trait, with the corresponding p-value shown in parentheses below. The color scale on the right ranges from blue (-1) to red (+1), representing negative to positive correlations, respectively. **(C)** Correlation network between genes and metabolites related to flavonoid, sugar, and acid metabolism in the turquoise module. Orange circles represent metabolites, and turquoise octagons represent genes. Positive regulatory relationships are illustrated with red edges, while negative ones are marked in blue.

Using Cytoscape software, a correlation network was constructed between DEGs in flavonoid, sugar, and organic acid metabolic pathways within the turquoise module and metabolite contents (**Figure 3C**). In total, 13 key genes exhibited significant correlations with metabolites, suggesting that they play important roles in metabolite accumulations in CM and BM fruit.

### 3.6 Key transcription factors and co-expression networks

A heatmap was used to display the expression profiles of all TFs in the turquoise module. A total of 29 TFs were identified in this module, most of which belonged to the ERF, MYB, and WRKY families. These TFs are closely associated with the targeted metabolites, suggesting that they play crucial regulatory roles in the flavonoid, sugar, and organic acid metabolic pathways (**Figure 4A**). To elucidate the transcriptional regulatory mechanisms of the flavonoid, sugar, and organic acid metabolic pathways in blueberry fruit, cis-acting elements in the promoter regions of 13 key genes were predicted (**Figure 4B**). Various types of cis-regulatory elements were enriched in the promoter regions, mainly including binding sites of MYB (MYB, MBS, and MBSI), bHLH (MYC), and WRKY (W-box) families, as well as hormone/stress response elements (ERE and DRE).

**Figure 4:**
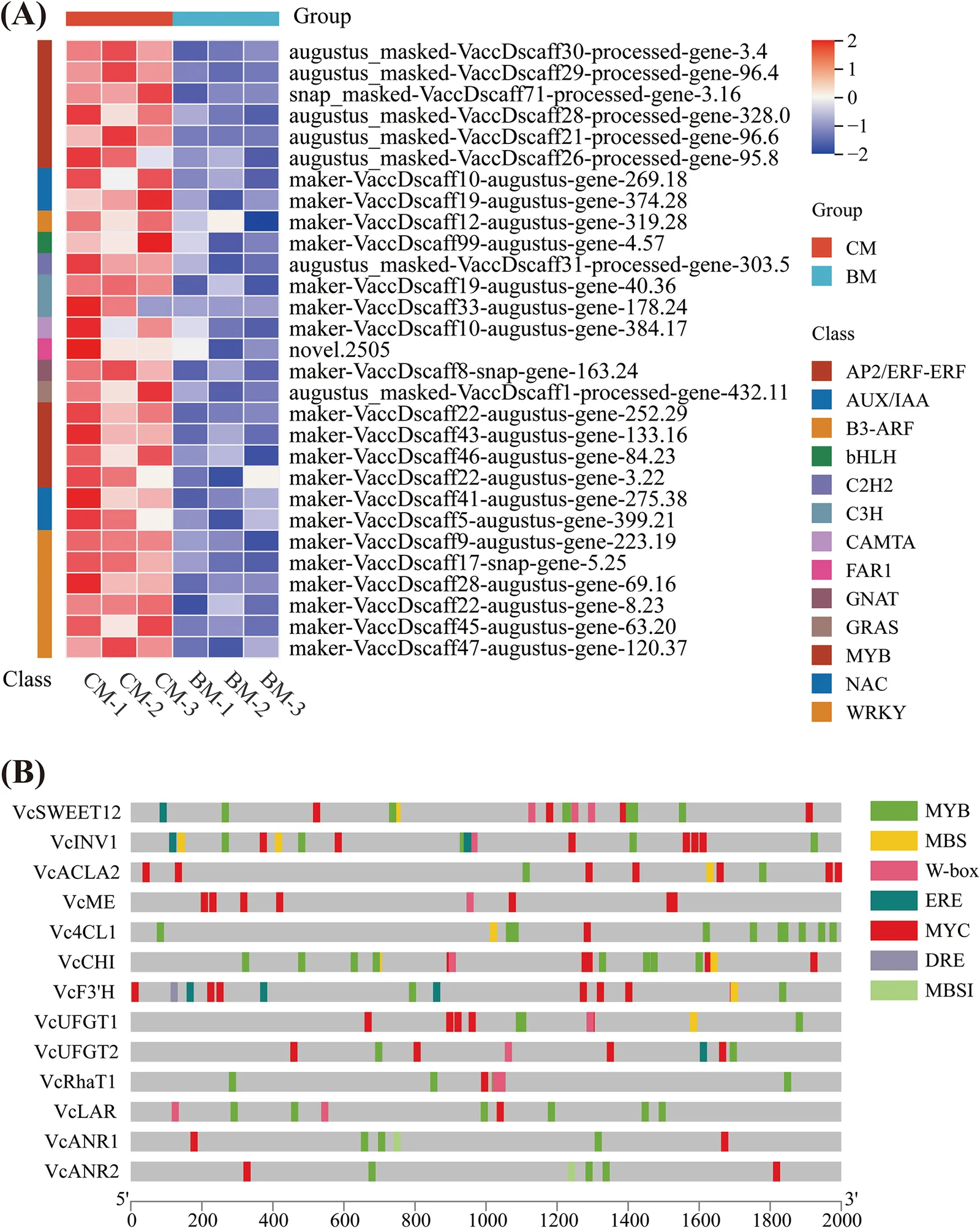
Analysis of expression patterns of transcription factors in key WGCNA module and cis-acting elements in the promoters of candidate key genes. **(A)** Expression profiles of transcription factors from the WGCNA hub module associated with fruit quality; **(B)** Distribution of cis-acting elements in the promoter sequences of selected key genes.

Owing to the cis-acting element analysis, TFs belonging to the MYB, WRKY, ERF, and bHLH families were screened from the turquoise module. Combined with the expression data of the 13 key genes, a Pearson’s correlation analysis was performed between TFs and target genes (**Supplementary Table S10**). The TFs with the highest correlation coefficients and predicted-binding sites in the promoters of target genes were selected. In total, nine core TFs were identified, *VcERF5-2*, *VcERF5-1*, *VcWRKY40*, *VcMYB36*, *VcWRKY24*, *VcWRKY53*, *VcWRKY75*, *VcbHLH35*, and *VcMYB5*. Using these nine screened core TFs, a co-expression network of correlations among TFs, target genes, and metabolites was constructed. These TFs were significantly correlated with target genes related to carbohydrate metabolism, the TCA cycle, and flavonoid biosynthesis. Additionally, they exhibited significantly positive correlations with flavonoid metabolites, such as epicatechin, and quercetin, as well as sugars, including glucose and fructose, and they exhibited significantly negative correlations with organic acids, such as malic acid and citric acid (**Figures 5A, B**).

**Figure 5:**
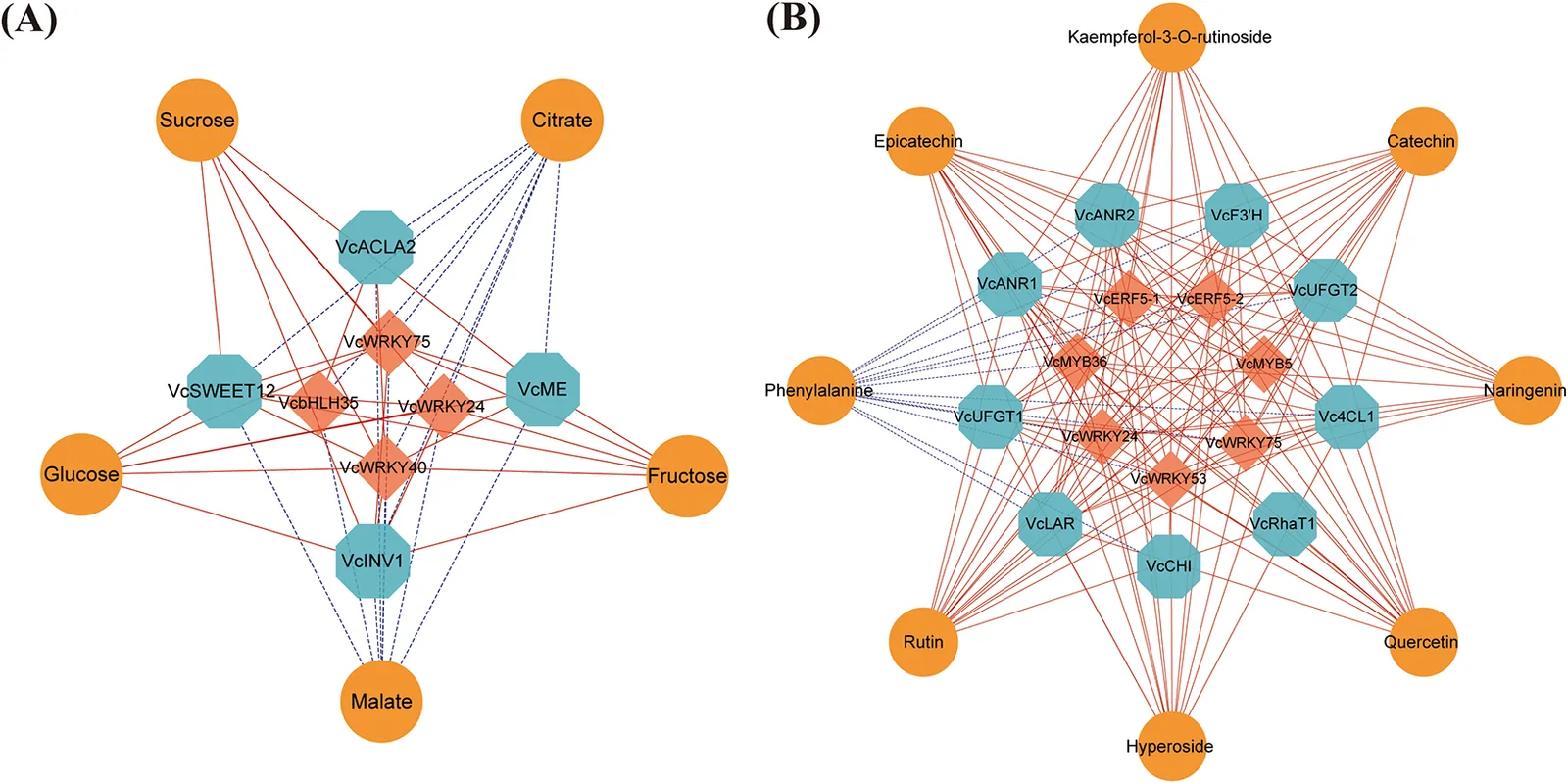
Correlation co-expression network of transcription factors (TFs) and candidate genes. **(A)** Correlation co-expression network of TFs and genes related to sugar and acid metabolites; **(B)** Correlation co-expression network of TFs and structural genes related to flavonoid metabolites. Orange circles represent metabolites, turquoise octagons represent candidate genes, and red diamonds represent TFs. Positive regulatory relationships are illustrated with red edges, while negative ones are marked in blue.

### 3.7 Validation of candidate genes by qRT-PCR

To verify the expression levels of candidate genes involved in flavonoid, sugar, and organic acid metabolic pathways, a qRT-PCR analysis was performed. In total, 20 DEGs were selected, 4 DEGs related to sugar and organic acid metabolism, 8 DEGs involved in flavonoid biosynthesis, and 8 TFs. As shown in **Figures 6A, B**, the RNA sequencing (RNA-seq) data were generally consistent with the qRT-PCR data. The log_2_FC values detected by qRT-PCR and RNA-seq exhibited a strongly positive correlation (Pearson’s correlation analysis, *r* = 0.7851, *p* < 0.0001), indicating a high consistency between the two detection methods and verifying the reliability of the transcriptome sequencing data.

**Figure 6:**
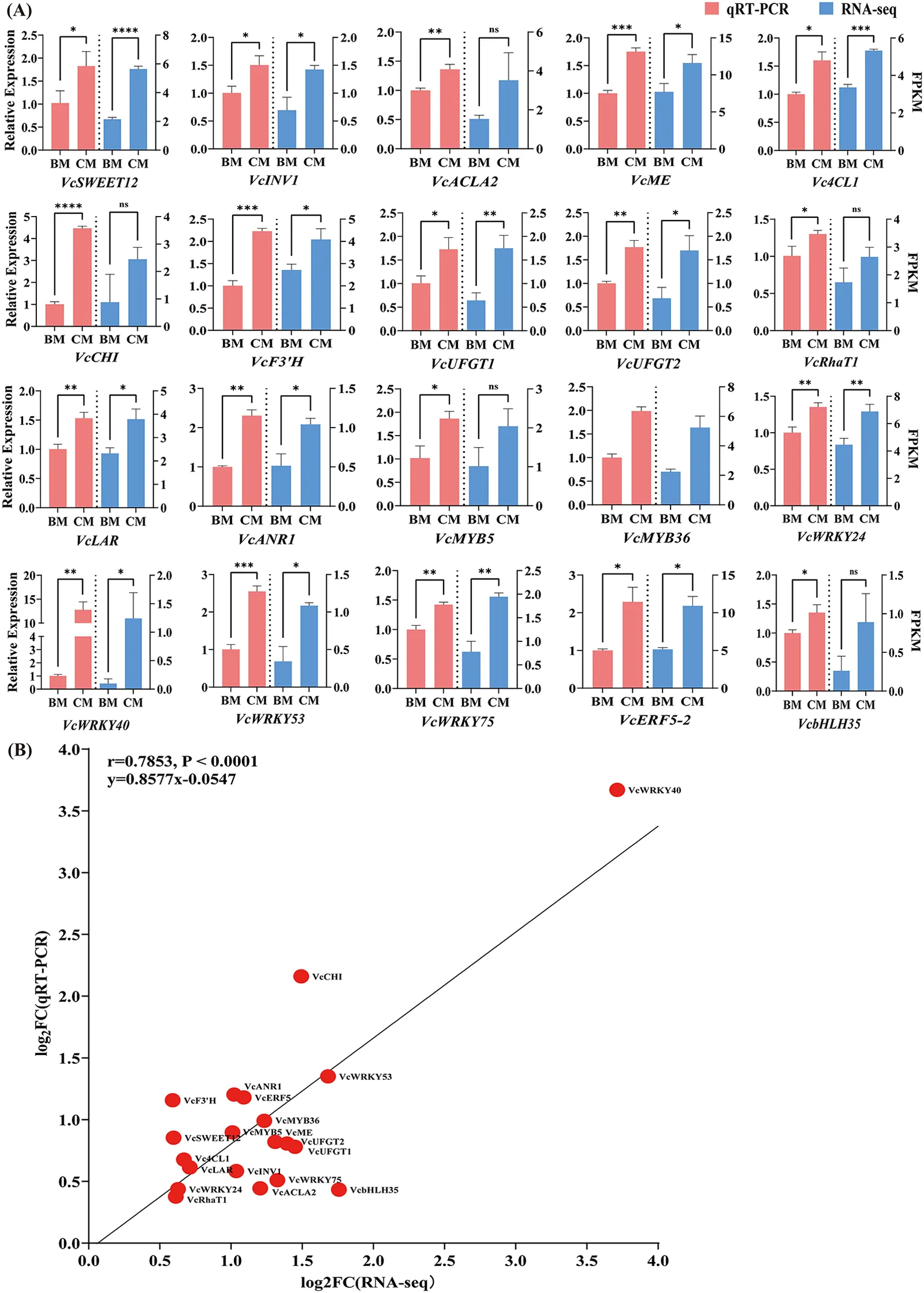
RNA-seq and qRT-PCR analysis of key candidate genes and transcription factors. **(A)** Validation and expression analysis of selected genes by qRT-PCR. The left y-axis represents the relative expression level of genes; the right y-axis represents the FPKM values obtained from RNA-seq. Data are presented as mean ± SE. *, **, *** and **** indicate significant differences at p < 0.05, 0.01, 0.001 and 0.0001 levels, respectively. **(B)** Scatter plot of the correlation between qRT-PCR and RNA-seq fold changes.

## 4 Discussion

### 4.1 Fruit quality

Selecting an appropriate rootstock–scion combination is fundamental for improving the yields of grafted trees, as rootstock–scion interactions affect water and mineral transport, leaf gas exchange, plant size, flowering and fruiting time, and fruit quality (Gonçalves et al., 2003).The results of this study showed that, compared with the ‘Brightwell’ rootstock, the ‘Climax’ rootstock promoted the expansion of ‘Misty’ blueberry fruit. Similarly, compared with a sour orange rootstock, a rough lemon rootstock increases the fruit weight, and the length and diameter, of the ‘Gunda Gundo’ orange (Aregay et al., 2021). Results of both the fuzzy comprehensive evaluation and PCA showed that the comprehensive fruit quality CM was superior to that of BM. Rootstock selection plays key roles in regulating sugar and acid metabolism, thereby influencing fruit flavor quality. The fruit quality of ‘Newhall’ navel orange grafted onto trifoliate orange rootstock is better than on red tangerine rootstock, which in turn is superior to sweet orange (Ma et al., 2025). Compared with trifoliate orange rootstock, the dwarfing rootstock ‘Flying Dragon’ increases the fruit size and sugar content of ‘Minihyang’ citrus, and the contents of fructose and sucrose in mature fruit of ‘Minihyang’ grafted onto ‘Flying Dragon’ rootstock are significantly greater than those grafted onto trifoliate orange (Hong et al., 2022). Likewise, fruit yield, morphological size, firmness, and the contents of sugars, organic acids, phenols, and other compounds in sweet cherry are affected by factors such as rootstock (Ozturk et al., 2025; Wang et al., 2025).

### 4.2 Photosynthetic characteristics and mineral nutrients

Photosynthesis is a key factor affecting the carbohydrate supply to fruit. Compared with the ‘Brightwell’ rootstock, the ‘Climax’ rootstock increased the *P_n_* of ‘Misty’ blueberry leaves, accompanied by synchronous increases in *G_s_*, *C_i_*, and *T_r_*.* A* decrease in *P_n_* accompanied by synchronous reductions in *C_i_* and *G_s_* indicated that the decline in photosynthesis may be partly associated with improved stomatal conductance (He and Wang, 2019). Thus, the improvement in *P_n_* of CM was partly attributed to stomatal limitation, which was also consistent with the results of the correlation analysis. In addition, chlorophyll a, chlorophyll b and total chlorophyll contents in CM leaves were significantly higher than those in BM leaves. Chlorophyll content is related to the light reaction, i.e., the absorption, transfer, and conversion of light energy, whereas stomatal limitation involves the CO_2_ supply for the carbon reaction. Therefore, the factors that promote the increase of *P_n_* in CM leaves include not only stomatal limitations, but also non stomatal factors such as chlorophyll.

Mineral nutrients serve as an important material bases for plant growth, development, and fruit quality formation. Studies on fruit crops have demonstrated that rootstocks substantially modify mineral nutrient uptake and accumulation in scions. Apple rootstocks modulate tree vigor, yield and fruit quality by altering total mineral element contents in scion cultivars (Amiri et al., 2014). Similarly, peach rootstocks change nutrient acquisition and partitioning in leaves and fruits (Bien-Aime and Melgar, 2025), and rootstock-dependent variation in leaf mineral profiles has also been reported in grafted SHB (Yu et al., 2024).

Recent studies further indicate that rootstock-regulated mineral nutrition contributes to fruit quality. Kaleem et al. (2026a) found pumpkin rootstocks altered N, P, and K uptake and allocation in melon; greater K partitioning to scions and fruits coincided with elevated soluble solids and soluble sugars. Li et al. (2024a) likewise reported that citrus rootstocks alter nitrogen absorption, distribution, and utilization, accompanied by divergent fruit quality. Collectively, these findings imply that rootstock-triggered shifts in mineral nutrition represent a key physiological factor shaping fruit quality. The results of the present study showed that, except for a lower leaf B content in CM than in BM, the leaf contents of K, Ca, Fe, Mn, Cu, and Zn were all higher in the CM. These differences indicate distinct nutritional statuses of the ‘Misty’ scion grafted onto the two rootstocks. Notably, CM also exhibited higher photosynthetic performance and improved fruit quality, including higher total soluble solids. Although the present data cannot confirm causal links between individual mineral elements and fruit quality, these coordinated changes suggest that rootstock-modulated mineral nutrition partly underpins the differences in scion performance and fruit quality (Milošević et al., 2023).

According to the optimal nutrient ranges for SHB in the USA (Bryson et al., 2014), the leaf contents of Ca, Mg, and Mn in CM and BM were relatively low, and the leaf Cu content of BM was also below the optimal range. The other elements were within optimal or high ranges. Likewise, the leaf contents of Ca, Mg, and Mn in ‘Emerald’ southern highbush blueberry grafted onto ‘Essence’ and ‘Baldwin’ rootstocks, as well as leaf B and Cu contents in ‘Emerald’ on ‘Essence’ rootstock, are at relatively low levels (Yu et al., 2024). The relative deficiencies of Ca, Mg, and Mn may impair the light reaction (Valverdi and Kalcsits, 2021a, 2021b; Worbington et al., 2025). Therefore, Ca, Mg, Mn and Cu nutrition merits close attention for field cultivation of grafted ‘Misty’ blueberry in this region, though targeted nutrient-management trials are required to define site-specific fertilization regimes.

### 4.3 Sugars and organic acids

Sugars, organic acids, and their ratio are core indicators of blueberry fruit flavor. The main soluble sugars in mature fruit of CM and BM were glucose, fructose and sucrose. The contents of these three sugars in CM fruit were significantly greater than in BM fruit. Transcriptome data showed that genes *SWEET12* and *INV1* were significantly upregulated in CM vs. BM. This facilitated sugar transport and sucrose conversion into glucose and fructose (**Supplementary Figure S1**), which was consistent with the quantitative determination results. Rootstock-dependent transcriptional changes in sugar metabolism are widely documented in grafted horticultural crops. In melon, pumpkin rootstock ‘Tianzhen No. 1’ upregulated the expression of sugar-transport and metabolism-related genes including *HT2*, *HT7*, *SWEET4*, *SWEET7*, *SPS1*, and *NIN3*, which coincided with increased soluble sugar accumulation in fruits (Kaleem et al., 2026a). In ‘Newhall’ sweet orange, rootstocks types altered the fruit developmental expression patterns of the acid/neutral invertase gene *CsA/N-InvA* and sucrose transporter genes *CsSUC3* and *CsSUC4* (Ma et al., 2025). These findings are highly consistent with our present results, in which rootstock variation induced differential expression of sugar metabolism-related genes *VcSWEET12* and *VcINV1* in blueberry. In this study, the contents of citric, malic, fumaric, and maleic acids were relatively high in BM fruit compared to CM. Citric, malic, and fumaric acids are key intermediates in the TCA cycle (Liu et al., 2022), whereas maleic and fumaric acids are isomers. Transcriptome data showed that in the CM vs. BM comparison, *PDH* was downregulated and *ACL* was upregulated, while *KDH*, *ME*, and *MDH* showed mixed expression, suggesting that the two rootstocks differentially regulate organic acid metabolism in the grafted ‘Misty’ scion and thereby contribute to the distinct organic acid accumulation in CM and BM fruit. Comparable rootstock effects on fruit acidity have been reported in citrus. Rootstock–scion combinations influenced acid degradation together with other fruit quality traits (Li et al., 2024b); rootstock-dependent changes in scion gene expression were associated with acid degradation during blood orange ripening (Lana et al., 2021); and different rootstocks of ‘Newhall’ sweet orange altered the expression of *CsCS1* and *CsNADP-IDH1/2/3*, paralleling differences in fruit organic acid accumulation (Ma et al., 2025). Together, these studies support a role for rootstock-dependent transcriptional regulation in fruit organic acid metabolism.

### 4.4 Amino acids and flavonoids

Amino acids and their derivatives are important components of blueberry fruit quality. Among the 28 detected amino acids, the contents of 26 amino acids were significantly higher in BM fruit than in CM fruit. This indicated that ‘Brightwell’ rootstock may be more conducive to the accumulation or biosynthesis of amino acids in ‘Misty’ blueberry. Rootstock-dependent differences in fruit amino acid composition have also been reported in ‘Newhall’ sweet orange (Xiong et al., 2023). Flavonoids are core nutritional components of blueberry fruit, with strong health benefits such as antioxidant and anti-inflammatory activities (Nicoletti et al., 2015; Lu et al., 2021; Jurja et al., 2025). In this study, CM exhibited a distinct advantage in flavonoid accumulation. Among the 31 detected flavonoids, 24 flavonoids had significantly greater contents in CM fruit than in BM fruit, including hyperoside, catechin, and other major flavonoids. Transcriptome sequencing showed that the expression levels of key flavonoid biosynthesis genes (*CHI*, *F3’H*, *UFGT*, *RhaT*, *LAR*, and *ANR*) were significantly upregulated in CM relative to BM (**Figure 2**). These results indicated that the presence of the ‘Climax’ rootstock was highly favorable for the biosynthesis and accumulation of most flavonoids in ‘Misty’ blueberry fruits. Similarly, when two blood orange cultivars were grafted onto eight different rootstocks, differences were detected in the contents of cinnamic acids and anthocyanin-related flavanones (Morales et al., 2021). Rootstock-scion combinations also affected anthocyanins, flavonols, flavanols, and flavanones in sweet cherry fruit (Boskov et al., 2023). Gene-level evidence further supports rootstock-dependent transcriptional regulation of flavonoid metabolism. In grapevine, hetero-grafting onto different rootstocks altered the expression of *VvUFGT1*, *VvLAR1*, *VvLAR2*, and *VvANR*, accompanied by changes in flavonoid accumulation (Zhang et al., 2022). Similarly, in ‘Newhall’ sweet orange, different rootstocks affected the expression of multiple flavonoid biosynthesis genes, including *CHI, F3’H, LAR*, and *ANR*, together with differences in flavonoid accumulation (Li et al., 2023b). These findings are consistent with the rootstock-associated changes in *VcCHI*, *VcF3’H*, *VcUFGT*, *VcLAR*, and *VcANR* observed in the present study. The observed pattern, in which 26 amino acids accumulated at higher levels in BM fruit while most flavonoids were more abundant in CM fruit, indicates a decoupling between nitrogen metabolism and flavonoid biosynthesis. This may reflect a shift in carbon and nitrogen allocation within the scion under different rootstock conditions.

### 4.5 Transcription factors

In this study, two candidate WRKY TFs, *VcWRKY75* and *VcWRKY24*, were identified. They showed significantly positive correlations with the key sugar metabolism genes *VcSWEET12* and *VcINV1*, respectively, and predicted W-box elements (specific binding motifs of the WRKY family) were present in the promoter regions of these target genes. These results suggested that *VcWRKY75* and *VcWRKY24* may positively regulate the expression of *VcSWEET12* and *VcINV1*, respectively, and thus participated in the regulation of sugar transport and sucrose decomposition. WRKY-mediated regulation of sugar metabolism has been widely reported in fruit crops, with WRKY TFs targeting SWEET, SUT, SUS, SPS, and INV genes to modulate sugar transport, accumulation, and sucrose metabolism (Li et al., 2020; Chen et al., 2022; Sun et al., 2022; Guan et al., 2025; Wu et al., 2025). Notably, *WRKY75* homologs have been associated with SWEET expression and sucrose accumulation (Sun et al., 2022), supporting the potential roles of *VcWRKY75* and *VcWRKY24* in regulating *VcSWEET12* and *VcINV1*, respectively.

Flavonoid biosynthesis is coordinately regulated by multiple TF families, particularly WRKY, ERF, and MYB. In this study, seven TFs (*VcWRKY24*, *VcWRKY75*, *VcWRKY53*, *VcERF5-1*, *VcERF5-2*, *VcMYB5*, and *VcMYB36*) were associated with key genes involved in flavonoid biosynthesis and modification. This regulatory pattern is consistent with previous evidence that WRKY TFs target structural genes such as *CHI*, *LAR*, and *UFGT* to promote flavonoid and proanthocyanidin accumulation (Wang et al., 2018; Chen et al., 2023; Zhao et al., 2024), whereas ERF TFs regulate anthocyanin biosynthesis through *UFGT* and *F3′H* and related pathway genes (Ma et al., 2021; Xu et al., 2025). MYB TFs likewise modulate flavonoid metabolism by targeting structural genes, including *4CL* (Song et al., 2023; Huang et al., 2025). Together, these findings suggest a coordinated WRKY–ERF–MYB regulatory network underlying flavonoid accumulation and modification in blueberry fruit. In this study, nine candidate TFs were identified to be closely correlated with the expression of key genes governing sugar, organic acid, and flavonoid metabolism in blueberry fruit. Integrating these correlation analyses with promoter *cis*-element predictions, we further proposed a hypothetical transcriptional regulatory model. This model illustrates the potential regulatory relationships underlying the differential accumulation of sugars and flavonoids in ‘Misty’ fruit grafted onto ‘Climax’ and ‘Brightwell’ rootstocks (**Figure 7**).

**Figure 7:**
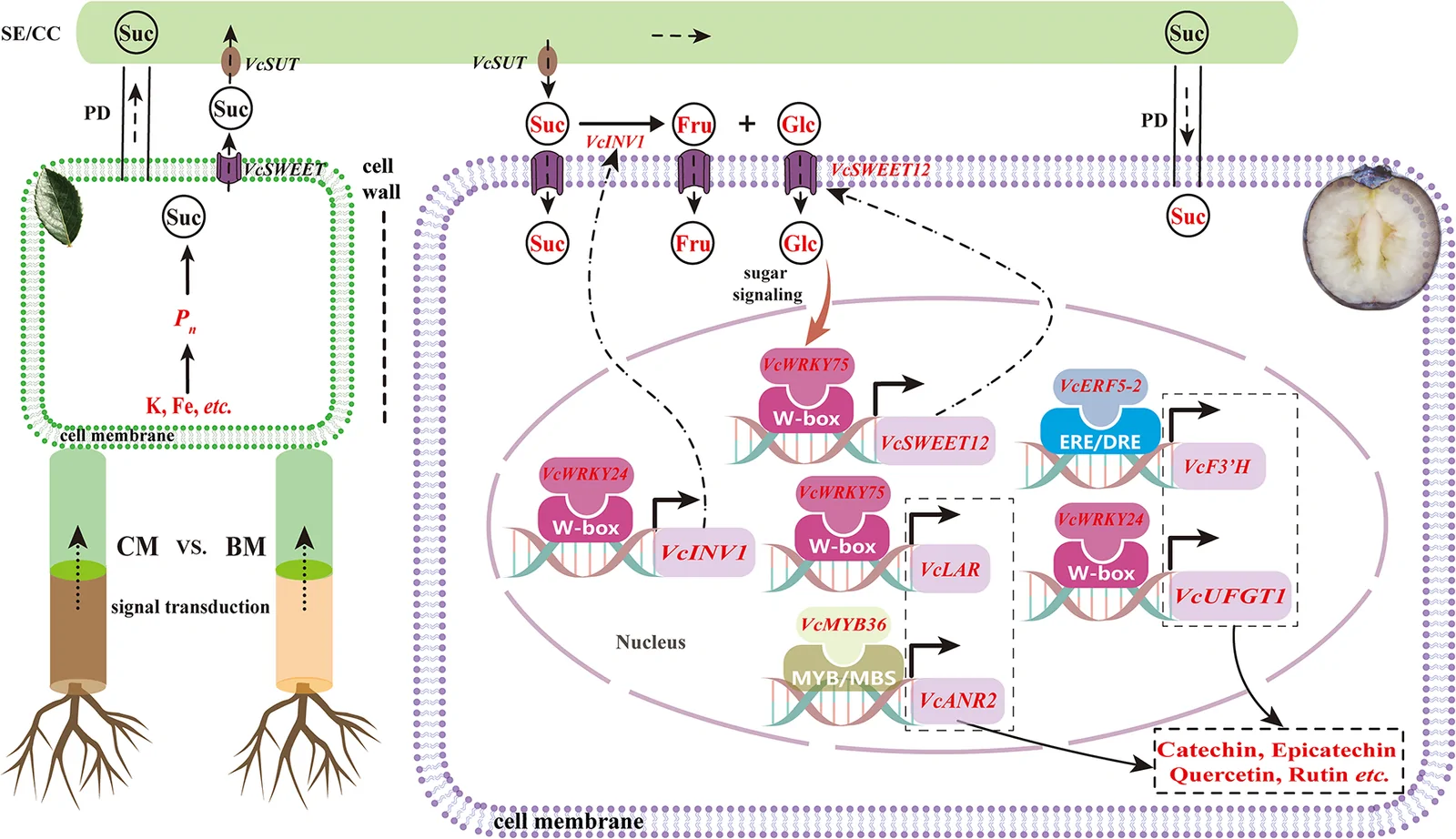
A hypothetical model of influence of rootstock grafting on sugars and flavonoids differential accumulation in scion fruit. CM, ‘Misty’ blueberry grafted onto ‘Climax’; BM, ‘Misty’ blueberry grafted onto ‘Brightwell’; K, potassium; Fe, iron; *P_n_*, net photosynthetic rate; PD, plasmodesmata; SE/CC, sieve element/companion cell; Glu, glucose; Fru, fructose; Suc, sucrose; SWEET, sugar transporter; SUT, sucrose transporter; INV, invertase; F3’H, flavonoid 3’-hydroxylase; LAR, leucoanthocyanidin reductase; ANR, anthocyanidin reductase; UFGT, flavonoid 3-O-glucosyltransferase; Red fonts indicate upregulated gene expression or increased metabolite accumulation in CM compared to BM.

Several limitations of the present study should be acknowledged. The predicted TF–target relationships remain to be functionally validated using approaches such as yeast one-hybrid, electrophoretic mobility shift assay, and dual-luciferase reporter assay. In addition, evaluation across additional growing environments would also help determine the stability and applicability of rootstock effects.

## 5 Conclusions

Compared with ‘Brightwell’, the ‘Climax’ rootstock was more conducive to improving the comprehensive quality of ‘Misty’ blueberry fruit. The ‘Climax’rootstock significantly promoted the *P_n_* in scion leaves and the accumulation of 4 sugars (glucose, fructose, sucrose, and maltose) and 24 flavonoids in scion fruit. While most amino acids and organic acids were more abundant in BM fruit. Genes associated with sugar (*VcSWEET12* and *VcINV1*) and flavonoids (*VcCHI, VcF3’H, VcUFGT*, *VcLAR*, and *VcANR*) were upregulated in CM fruit. In addition, core TFs, including VcWRKY75, VcWRKY24, VcbHLH35, VcWRKY40, VcWRKY53, VcERF5-1, VcERF5-2, VcMYB5, and VcMYB36, were identified. They were associated with the expression of key genes regulating sugar, organic acid, and flavonoid metabolism. These identified candidate TFs and genes can be used to guide blueberry breeding and management strategies that improve fruit quality and soil adaptability during blueberry production.

## Data Availability

The datasets presented in this study can be found in online repositories. The names of the repository/repositories and accession number(s) can be found below: https://www.ncbi.nlm.nih.gov/, PRJNA1455170.
