# Supplementary material for: Hetero-grafting affects sugar and flavonoid accumulation in ‘Misty’ blueberry (Vaccinium corymbosum)
Source: Front Plant Sci. 2026 Oct 22;17:1995904. doi: 10.3389/fpls.2026.1995904 (PMC13735575; doi:10.3389/fpls.2026.1995904)
Supplement: Supplementary file 1 [file SupplementaryFile1.docx]

**Table S1.** Primer Sequences of Differentially Expressed Genes

| gene ID | gene name | forward primer(5’→3’) | reverse primer(5’→3’) |
| --- | --- | --- | --- |
|  | *GAPDH* | CGGCTACTTACGAGCAAATCAA | TTCAGTGTAGCCCAAAATTCCTTT |
| *maker-VaccDscaff7-augustus-gene-231.27* | *VcSWEET12* | ACGCACCGAAGAAATCGA | AAGGCAAATCCACCCAACT |
| *maker-VaccDscaff34-augustus-gene-17. 21* | *VcINV1* | CAATGAGTCCACCAATGCA | CCAACTTTGTTCACCCGTAG |
| *maker-VaccDscaff2-augustus-gene-334.29* | *VcACLA2* | TTGGAGACTTCATAATGGGC | ATTCACCAGGGTAAACGGA |
| *maker-VaccDscaff36-augustus-gene-42. 34* | *VcME* | GACAAATCGGTCGTAGGCG | AGACAGTCCAAGGGGTAACAA |
| *augustus_masked-VaccDscaff43-processed-gene-132.9* | *Vc4CL1* | GCAAAATAGTTGGCGTAGGA | AAGTCTGGTTTCTTGGGGAT |
| *maker-VaccDscaff633-augustus-gene-0. 8* | *VcCHI* | ATGGATACAGTTGGAAGTCCAGAAT | ATTCTGGACTTCCAACTGTATCCAT |
| *maker-VaccDscaff42-augustus-gene-190. 35* | *VcF3'H* | CAACGGACACTACAGCAACC | GTGGGACTCTTCGACAACCT |
| *maker-VaccDscaff5-augustus-gene-295. 34* | *VcUFGT1* | CCACCCAATCGCTACTACTCA | GACAAAATTCAACCGCTCCT |
| *novel.84* | *VcUFGT2* | GTTGAAGGACTCGGACGCT | TCCAGTACCCTAGAAGGCAATA |
| *maker-VaccDscaff39-augustus-gene-69. 40* | *VcRhaT1* | ATATTACCAGTGGGCCTACTTC | CCGATGATTTCTTTTGCTTG |
| *maker-VaccDscaff25-augustus-gene-293. 25* | *VcLAR* | GCGAACTCTACCCAGATGAA | CGGTTACGCCTATGCTTTT |
| *maker-VaccDscaff19-augustus-gene-256. 19* | *VcANR1* | AAGACCCTAGCGGAGAAAGC | GGGAGAAACACCAGCCATAA |
| *maker-VaccDscaff22-augustus-gene-3. 22* | *VcMYB5* | GGCGGCGATAACAATCTAG | AGCAACGACTCAGCAAAGG |
| *maker-VaccDscaff22-augustus-gene-252.29* | *MYB36* | ATGGGGAGGGTTCCCTGCTGTGACA | TGTCACAGCAGGGAACCCTCCCCAT |
| *maker-VaccDscaff47-augustus-gene-120.37* | *VcWRKY24* | CAGATTCCCAAAGCCACG | TTTCACTCCCTTTCACTTGC |
| *maker-VaccDscaff28-augustus-gene-69. 16* | *VcWRKY40* | TAGCAGTTCCAAATCTCCAAAG | ACAAACACCTCCGCCATC |
| *maker-VaccDscaff22-augustus-gene-8. 23* | *VcWRKY53* | ATGGAAACTCTAATCAATGAGCTAA | TTAGCTCATTGATTAGAGTTTCCAT |
| *maker-VaccDscaff9-augustus-gene-223.19* | *VcWRKY75* | ATGGAGAACTACCCTTCAATGTTTC | GAAACATTGAAGGGTAGTTCTCCAT |
| *augustus_masked-VaccDscaff29-processed-gene-96. 4* | *VcERF5-2* | CTTTGCGGCTGGAGTGAT | GAGTAACCGAGCGTAGGGA |
| *maker-VaccDscaff99-augustus-gene-4. 57* | *VcbHLH35* | TCATTACTGCCAACATCACCG | GCTCACTGGGCTTTCTGGAT |

**Table S2.** Growth and Yield of ‘Misty’ Blueberry Grafted onto ‘Climax’ (CM) and ‘Brightwell’ (BM)

| rootstock-scion combination | tree height  (cm) | canopy diameter  (cm) | rootstock height  (cm) | rootstock diameter (cm) | scion diameter  (cm) | fruit yield（g/plant） |
| --- | --- | --- | --- | --- | --- | --- |
| CM | 136.60±1.15 | 136.32±9.34 | 21.33±1.55 | 4.99±0.40 | 123.42±8.71* | 2221.37±319.09 |
| BM | 138.28±0.98 | 141.23±5.24 | 21.75±0.77 | 4.30±0.35 | 101.60±2.47 | 1978.17±150.00 |

Note: Data are mean ± standard deviation (n = 3). * indicates significant difference between CM and BM according to independent-samples t-test (*p* < 0.05).

Table S3. Weight Values of Blueberry Fruit Traits

| trait | total soluble solids | fresh weight | anthocyanin | solid: acid ratio | titratable acidity | vitamin C | longitudinal  diameter | transverse  diameter | hardness |
| --- | --- | --- | --- | --- | --- | --- | --- | --- | --- |
| weight coefficient | 0.3 | 0.2 | 0.1 | 0.1 | 0.05 | 0.05 | 0.05 | 0.05 | 0.1 |

**Table S4.** Comprehensive Evaluation of Fruit Quality Traits of ‘Misty’ Blueberry Grafted onto ‘Climax’ (CM) and ‘Brightwell’ (BM)

| rootstock-scion combination | comprehensive evaluation value | the quality level |
| --- | --- | --- |
| CM | 0.729 | excellent |
| BM | 0.276 | medium |

**Table S5.** Characteristic Vector, Eigenvalue, Variance Contribution Rate, and Cumulative Contribution Rate of Two Principal Components

| index of quality | principal component 1 | principal component 2 |
| --- | --- | --- |
| fresh weight per fruit | 0.999* | -0.002 |
| hardness | 0.647* | -0.351 |
| longitudinal diameter | 0.935* | 0.307 |
| transverse diameter | 0.950* | 0.166 |
| titratable acid | 0.915* | 0.361 |
| total soluble solids | -0.137 | 0.960* |
| solid : acid ratio | -0.976* | -0.07 |
| vitamin C | 0.985* | -0.1 |
| anthocyanin | -0.524* | 0.734* |
| characteristic value | 6.248 | 1.852 |
| variance contribution rate (%) | 69.425 | 20.582 |
| cumulative contribution rate (%) | 69.425 | 90.007 |

Note: * represents the absolute value of correlation coefficient > 0.5.

**Table S6.** Principal Component Score and Comprehensive Evaluation of Mature Fruit of ‘Misty’ Blueberry Grafted onto ‘Climax’ (CM) and ‘Brightwell’ (BM)

| rootstock-scion combination | principal component score | | synthesis score | ranking |
| --- | --- | --- | --- | --- |
|  | P1 | P2 |  |  |
| CM | 5.67 | 0.06 | 3.95 | 1 |
| BM | -5.67 | -0.06 | -3.95 | 2 |

**T****able S7.** Correlation Between Photosynthetic Parameters in Leaves of ‘Misty’ Blueberry

| photosynthetic parameters | *P_n_* | *G_s_* | *C_i_* | *T_r_* |
| --- | --- | --- | --- | --- |
| *P_n_* | 1 |  |  |  |
| *G_s_* | 0.920** | 1 |  |  |
| *C_i_* | 0.927** | 0.960** | 1 |  |
| *T_r_* | 0.891** | 0.991** | 0.970** | 1 |

Note: Pearson correlation coefficients are shown (n = 6). * and ** mean significant correlation at the 5% and 1% levels, respectively.

**Table S8.** Mineral element contents in leaves of ‘Misty’ blueberry grafted onto ‘Climax’ (CM) and ‘Brightwell’ (BM)

| mineral elements | CM | BM |
| --- | --- | --- |
| P(g/kg) | 1.55±0.03 | 1.51±0.02 |
| K(g/kg) | 5.85±0.06* | 5.23±0.07 |
| Ca(g/kg) | 4.07±0.02* | 3.68±0.05 |
| Mg(g/kg) | 1.48±0.02 | 1.43±0.03 |
| Fe (mg/kg) | 326.25±2.55* | 277.09±4.33 |
| Mn(mg/kg) | 174.31±1.06* | 143.79±0.79 |
| Cu(mg/kg) | 7.18±0.10* | 3.52±0.04 |
| Zn(mg/kg) | 39.00±0.25* | 30.11±0.49 |
| B(mg/kg) | 24.57±0.21 | 27.84±0.45* |

Note: Data are mean ± standard deviation (n = 3), * indicates significant difference between CM and BM according to independent-samples t-test .

**Table S9.** Summary of Transcriptome Sequencing Data

| Sample | Raw Reads | Raw Base(G) | Clean Reads | Clean Base(G) | Error Rate(%) | Q30(%) | GC Content(%) |
| --- | --- | --- | --- | --- | --- | --- | --- |
| BM-1 | 61630666 | 9.24 | 59987130 | 9 | 0.02 | 97.27 | 46.31 |
| BM-2 | 55625176 | 8.34 | 53990194 | 8.1 | 0.02 | 97.54 | 46.35 |
| BM-3 | 59347554 | 8.9 | 57969432 | 8.7 | 0.02 | 97.48 | 46.45 |
| CM-1 | 59909584 | 8.99 | 58476484 | 8.77 | 0.02 | 97.62 | 46.41 |
| CM-2 | 55427590 | 8.31 | 53732562 | 8.06 | 0.02 | 97.34 | 46.33 |
| CM-3 | 52545892 | 7.88 | 50965022 | 7.64 | 0.02 | 97.35 | 46.42 |

**Table S10.** Correlation Analysis Between Structural Genes and Transcription Factors

| DEGs | TFs | TF classification | Correlation | p-value |
| --- | --- | --- | --- | --- |
| *VcSWEET12* | *maker-VaccDscaff9-augustus-gene-223.19(VcWRKY75)* | WRKY | 0.977029309 | 0.000785419 |
| *VcINV1* | *maker-VaccDscaff47-augustus-gene-120.37(VcWRKY24)* | WRKY | 0.95254279 | 0.003324839 |
| *VcACLA2* | *maker-VaccDscaff99-augustus-gene-4.57(VcbHLH35)* | bHLH | 0.882390747 | 0.019934523 |
| *VcME* | *maker-VaccDscaff28-augustus-gene-69.16(VcWRKY40)* | WRKY | 0.954337667 | 0.003079969 |
| *Vc4CL1* | *maker-VaccDscaff22-augustus-gene-252.29(VcMYB36)* | MYB | 0.959220837 | 0.002460504 |
| *VcCHI* | *maker-VaccDscaff22-augustus-gene-8.23(VcWRKY53)* | WRKY | 0.901188196 | 0.014163271 |
| *VcF3'H* | *augustus_masked-VaccDscaff29-processed-gene-96.4(VcERF5-2)* | ERF | 0.964668618 | 0.001850408 |
| *VcUFGT1* | *maker-VaccDscaff47-augustus-gene-120.37(VcWRKY24)* | WRKY | 0.980453113 | 0.000569387 |
| *VcUFGT2* | *augustus_masked-VaccDscaff21-processed-gene-96.6(VcERF5-1)* | ERF | 0.970846752 | 0.001262479 |
| *VcRhaT1* | *maker-VaccDscaff22-augustus-gene-252.29(VcMYB36)* | MYB | 0.819393535 | 0.045982469 |
| *VcLAR* | *maker-VaccDscaff9-augustus-gene-223.19(VcWRKY75)* | WRKY | 0.943556393 | 0.00468891 |
| *VcANR1* | *maker-VaccDscaff22-augustus-gene-3.22(VcMYB5)* | MYB | 0.937109974 | 0.005808363 |
| *VcANR2* | *maker-VaccDscaff22-augustus-gene-252.29(VcMYB36)* | MYB | 0.958979533 | 0.002489506 |

**Fig. S1.** Sugar-acid metabolism in mature fruit of ‘Misty’ blueberry grafted onto ‘Climax’ (CM) and ‘Brightwell’ (BM). SWEET, sugar transporter; INV, invertase; FK, fructokinase; PDH, pyruvate dehydrogenase complex; ACC, acetyl-CoA carboxylase；ACL, ATP-citrate lyase; KDH, α-ketoglutarate dehydrogenase complex; MDH, malate dehydrogenase; ME, malic enzyme. Red fonts indicate upregulated gene expression or increased metabolite accumulation in CM compared to BM, and blue fonts denote downregulation or reduced metabolite levels. The heatmap indicate the degree of regulation, with red representing upregulation, blue representing downregulation.
